# 

**Published:** 1896-08-01

**Authors:** 


**Notices of Births, Deaths, and Marriages** are inserted in this column at a charge of 2s. 6d. for an announcement not exceeding four lines.

### NOTICE TO CORRESPONDENTS.

All MS., Letters, Books for Review, and other matters intended for the Editor should be addressed The Editor, The Lodge, Porchester Square, London, W.

The Editor cannot undertake to return rejected MS., even when accompanied by stamped directed envelope.

**Notice to Advertisers and Subscribers.**

All Advertisements, Orders for Copies of The Hospital and Business Communications should be addressed to **THE MANAGER, (Not to The Editor),** The Hospital, 428, Strand, London, W.C.

The Cost of Subscription, post free, is as follows:—Per week, 2½d.; per quarter, 2s. 8d.; per half-year, 5s. 3d.; per annum, 10s. 6d. Foreign:—Per Annum, 15s. 2d.; payable, in all cases, in advance.

Price 5s.

**"Burdett's Hospitals and Charities, 1896."**

Seventh year of publication. Over 1,000 pp. Scarlet cloth, gilt lettered, Price 5s. A most complete guide to British, Colonial, Indian, and American Hospitals, Dispensaries, Nursing and Convalescent Institutions, and Asylums. A few complete sets of the preceding six issues of the "Annual" may still be obtained on application to the Publishers, The Scientific Press (Limited), 428, Strand, London, W.C.

**NOTICE.—Vol. XIX. of "The Hospital" (October, 1895, to April, 1896), in handsome cloth binding, is on sale at the Office of this Journal. Price 7s. 6d. Cases for binding the Numbers contained in this Volume, 1s. 6d. Index to Vol. XIX., 2½d., post free. The Monthly Part for August is now ready. Price 6d., by post 8d.**

### THE STUDENT OF MEDICINE.

### VACANCIES.

### Resident Medical Officers.

County Asylum, Shrewsbury.—Junior Assistant Medical Officer, not over 27 years of age. Salary to commence, £100 per annum (and £8 in lieu of beer, &c.), with board, lodging, and washing, rising to £120 at the end of two years' service. Applications to the Medical Superintendent at the Asylum by August 7th.

Cumberland Infirmary, Carlisle.—House-Surgeon. Appointment for one year. Salary £70 per annum, with board, lodging and washing. Applications to the Secretary by August 11th.

Earlswood Asylum for Idiots, Redhill, Surrey.—Medical Superintendent; aged 30 to 45. Salary £500 per annum, with furnished residence in the building, together with coals and gas. Applications, endorsed "Medical Superintendent," to be addressed to the Board of Management, 26, King William Street, London Bridge, E.C., by August 18th.

General Hospital, Nottingham.—House-Surgeon. Salary £100 per annum, rising £10 a year to £120. Applications to the Secretary by August 8th.

Miller Hospital and Royal Kent Dispensary, Greenwich Road, S.E.—Junior Resident Medical Officer. Salary £30 per annum, with board, attendance, and washing. Appointment tenable for six months, with prospect of re-election as Senior, at salary of £60. Applications to the Hon. Secretary or Secretary by August 6th.

Royal Eye Hospital or Royal South London Ophthalmic Hospital, Southwark.—House Surgeon; will be required to take up residence about October 1st. Salary £50 per annum, with board and lodging. Applications to the Secretary by August 1st.

Southport Infirmary.—Assistant House and Visiting Surgeon, doubly qualified. Honorarium at the rate of £80 per annum. Residence, board, and washing provided. Applications to Joseph Worrall, Infirmary Office, Southport, by August 14th.

Sussex County Hospital, Brighton.—Fourth Resident Medical Officer; doubly qualified, unmarried, and under 30 years of age. Salary £30 per annum, with board, washing, and residence in the hospital. Applications to the Secretary by August 5th.

### Non-Resident Medical Officers.

Central Provident Dispensary, Bedford.—Two Medical Officers. Applications to the Honorary Secretary by August 31st.

